# Supplementary material for: Interdependence between confirmed and discarded cases of dengue, chikungunya and Zika viruses in Brazil: A multivariate time-series analysis
Source: PLoS One. 2020 Feb 3;15(2):e0228347. doi: 10.1371/journal.pone.0228347 (PMC6996800; doi:10.1371/journal.pone.0228347)
Supplement: S1 Table — Brazil, January 2015 to December 2017. (PDF) [file pone.0228347.s001.pdf]

Table 1: Results of Dickey-Fuller Test applied to the series of confirmed and discarded cases of dengue, chikungunya and Zika before and after differentiation. Brazil, January 2015 to December 2017.

	Z_{1t}	Z_{2t}	C_{1t}	C_{2t}	D_{2t}	D_{2t}	DZ_{1t}	DZ_{2t}	DC_{1t}	DC_{2t}	DD_{2t}	DD_{2t}
Test Statistic	-2.25	-2.33	-2.36	-1.90	-4.29	-2.60	-3.68	-4.50	-4.92	-6.89	-5.34	-5.58
p-value	0.19	0.16	0.15	0.33	<0.001	0.09	0.004	<0.001	<0.001	<0.001	<0.001	<0.001
#Lags Used	12	8	6	2	6	10	11	7	5	1	10	9
Number of Observations Used	143	147	149	153	149	145	143	147	149	153	144	145
