# Supplementary material for: Interdependence between confirmed and discarded cases of dengue, chikungunya and Zika viruses in Brazil: A multivariate time-series analysis
Source: PLoS One. 2020 Feb 3;15(2):e0228347. doi: 10.1371/journal.pone.0228347 (PMC6996800; doi:10.1371/journal.pone.0228347)
Supplement: S2 Table — Brazil, January 2015 to December 2017. (PDF) [file pone.0228347.s002.pdf]

Table 1: AIC and Lag values for the restricts and unrestricted models for the series confirmed and discarded cases of dengue, chikungunya and Zika. Brazil, January 2015 to December 2017.

	Unrestricted model	Restricted model without Zika series	Restricted model without chikungunya series	Restricted model without Dengue series
AIC values	69.44	52.06	48.50	41.67
#Lags	13	12	13	12
