# Supplementary material for: Interdependence between confirmed and discarded cases of dengue, chikungunya and Zika viruses in Brazil: A multivariate time-series analysis
Source: PLoS One. 2020 Feb 3;15(2):e0228347. doi: 10.1371/journal.pone.0228347 (PMC6996800; doi:10.1371/journal.pone.0228347)
Supplement: S3 Table — Exploratory search of associations between series of confirmed and discarded cases of dengue, chikungunya and Zika. Brazil, January 2015 to December 2017. (PDF) [file pone.0228347.s003.pdf]

Table 1. Results of pairwise Granger tests for restricted models. Exploratory search of associations between series of confirmed and discarded cases of dengue, chikungunya and Zika. Brazil, January 2015 to December 2017.

Null hypothesis	Test statistic	p-value	Result
**Restricted model without Zika series**			
Discarded cases of chikungunya do not affect confirmed cases of dengue	2.284	0.008	Reject
Confirmed cases of dengue do not affect confirmed cases of chikungunya	0.7111	0.74	Do not reject
Confirmed cases of chikungunya do not affect confirmed cases of dengue	1.300	0.216	Do not reject
Confirmed cases of dengue do not affect discarded cases of chikungunya	0.9936	0.454	Do not reject
Discarded cases of dengue do not affect confirmed cases of chikungunya	0.7065	0.745	Do not reject
Confirmed cases of chikungunya do not affect discarded cases of dengue	1.417	0.155	Do not reject
**Restricted model without dengue series**			
Confirmed cases of Zika do not affect discarded cases of chikungunya	2.206	0.011	Reject
Discarded cases of Zika do not affect confirmed cases of chikungunya	2.447	0.004	Reject
Confirmed cases of Zika do not affect confirmed chikungunya	1.761	0.053	Do not reject
Confirmed cases of chikungunya do not affect confirmed cases of Zika	0.7291	0.723	Do not reject
Discarded cases of chikungunya do not affect confirmed cases of Zika	1.016	0.433	Do not reject
Discarded cases of Zika do not affect confirmed cases of chikungunya	0.8772	0.571	Do not reject
**Restricted model without chikungunya series**			
Confirmed cases of Zika do not affect discarded cases of dengue	4.377	< 0.001	Reject
Discarded cases of dengue do not affect confirmed cases of Zika	5.227	< 0.001	Reject
Confirmed cases of dengue do not affect discarded cases of Zika	5.479	< 0.001	Reject
Confirmed cases of Zika do not affect confirmed cases of dengue	6.442	< 0.001	Reject
Confirmed cases of dengue do not affect confirmed cases of Zika	5.227	< 0.001	Reject
Discarded cases of Zika do not affect confirmed cases of dengue	4.690	< 0.001	Reject
