# Supplementary figures and images for: Interdependence between confirmed and discarded cases of dengue, chikungunya and Zika viruses in Brazil: A multivariate time-series analysis
Source: PLoS One. 2020 Feb 3;15(2):e0228347. doi: 10.1371/journal.pone.0228347 (PMC6996800; doi:10.1371/journal.pone.0228347)

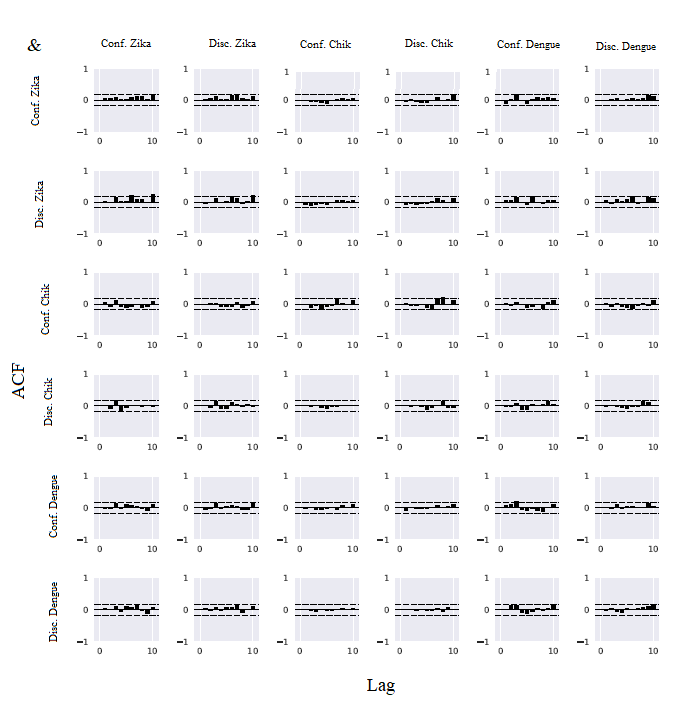

Supplement: S1 Fig — The plots along the diagonal are the individual ACFs for each model’s residuals, the remaining subplots show the cross-correlations between pairwise residuals. Here T is the sample size. The plots suggests randomness of the residuals, indicating a good fitting of the VAR model. (PNG) [file pone.0228347.s004.png]

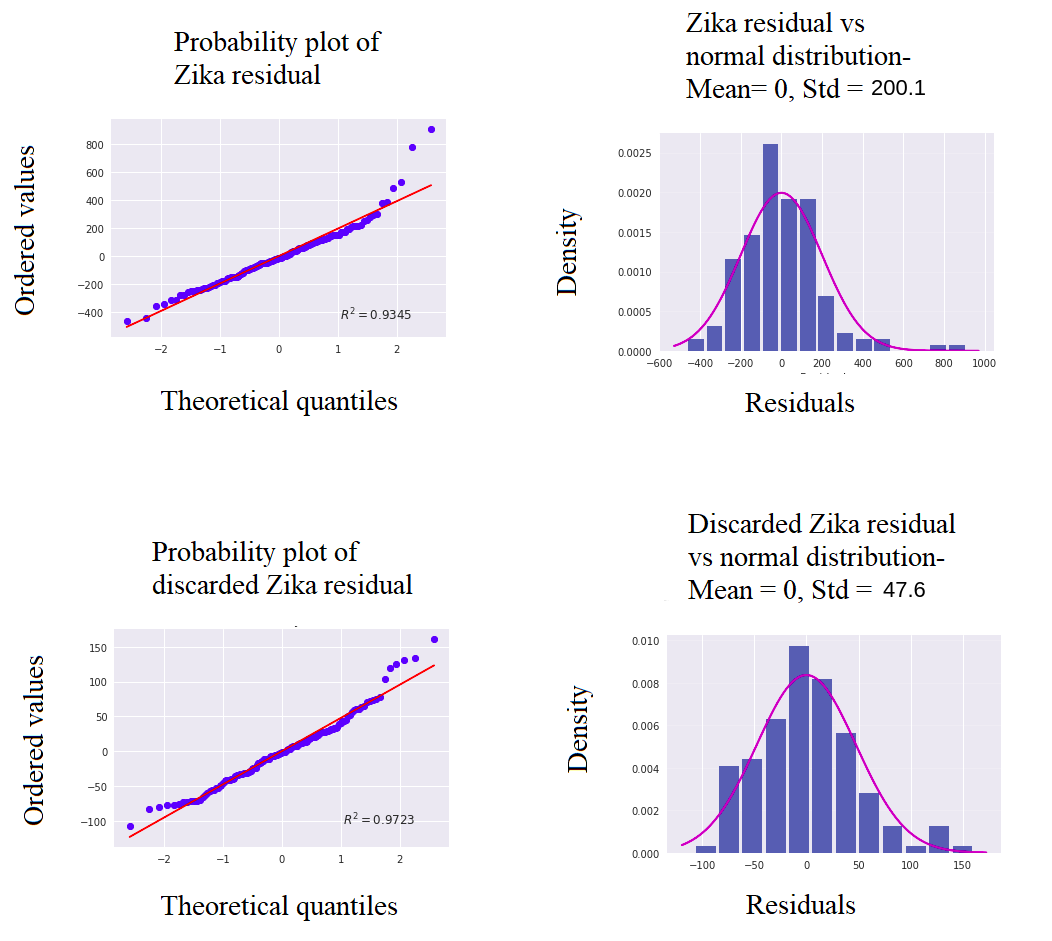

Supplement: S2 Fig — The probability plot shows the unscaled quantiles of residuals versus the probabilities of a normal distribution. (PNG) [file pone.0228347.s005.png]

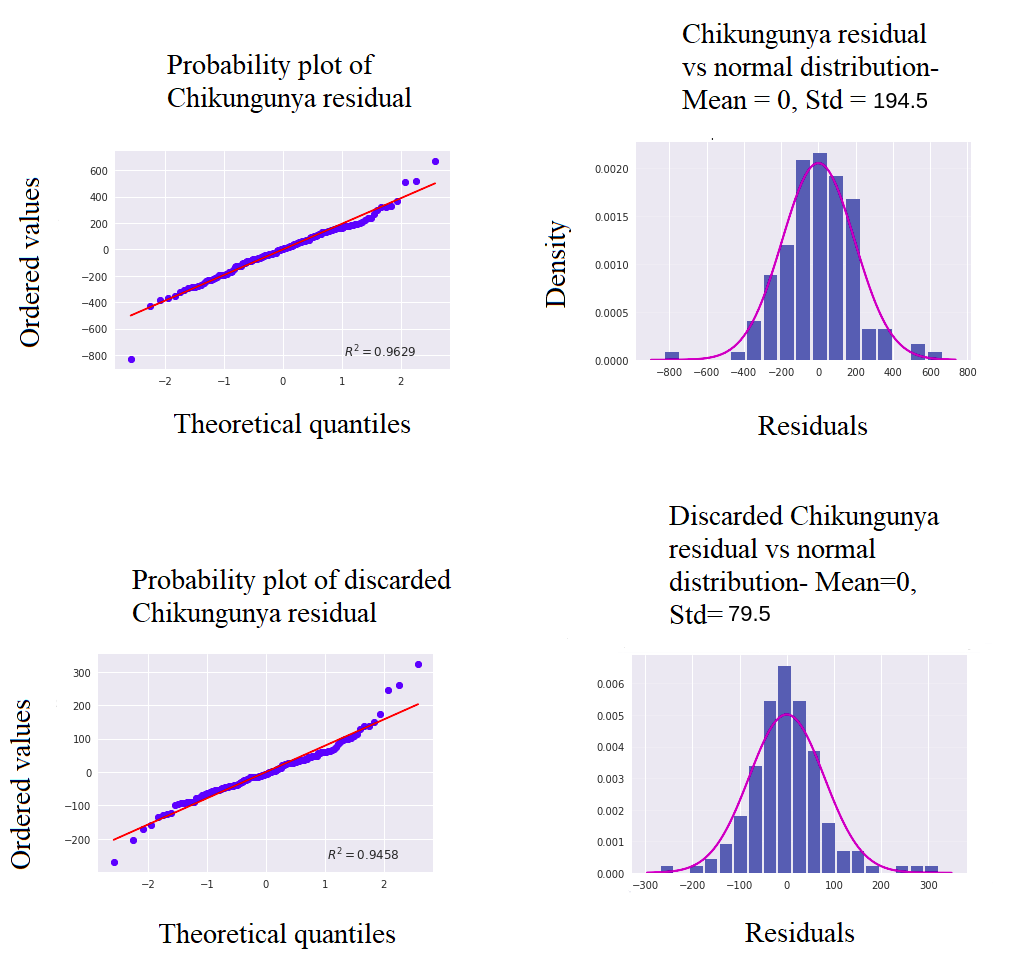

Supplement: S3 Fig — The probability plot shows the unscaled quantiles of residuals versus the probabilities of a normal distribution. (PNG) [file pone.0228347.s006.png]

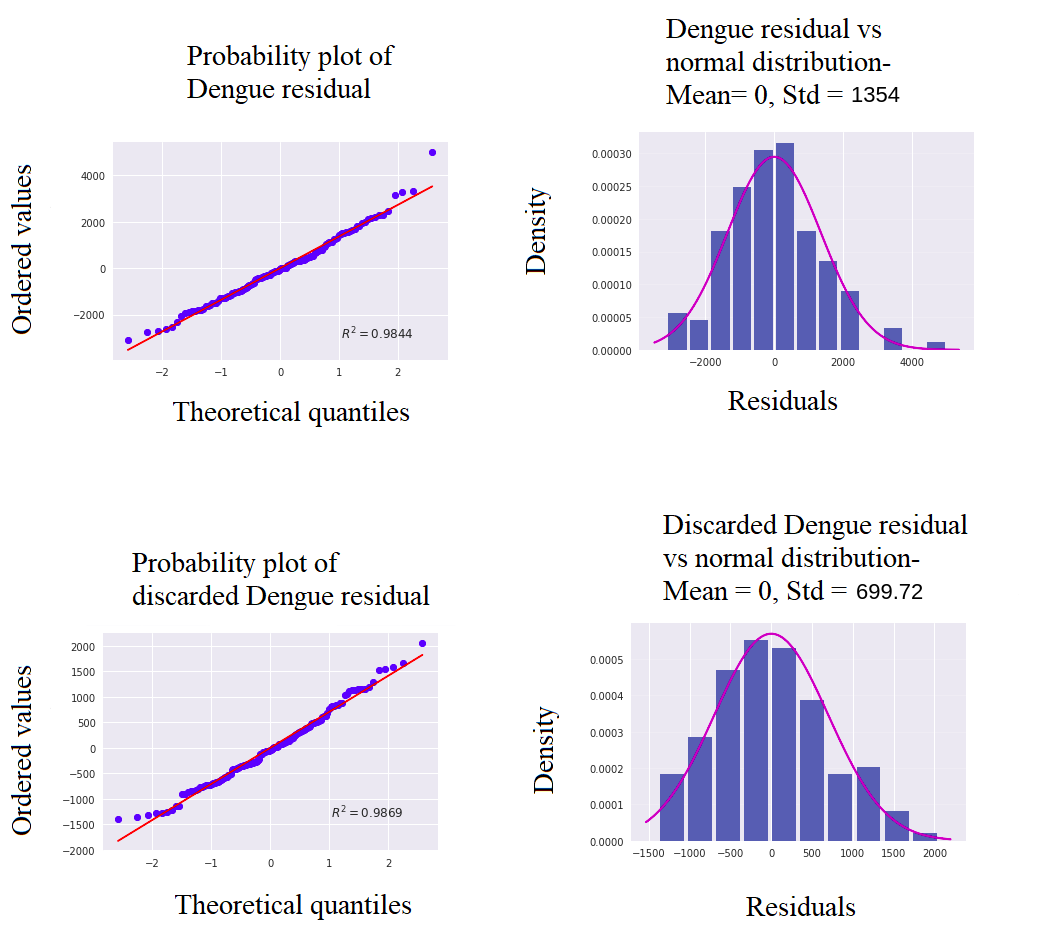

Supplement: S4 Fig — The probability plot shows the unscaled quantiles of residuals versus the probabilities of a normal distribution. (PNG) [file pone.0228347.s007.png]
