# Supplementary material for: Interdependence between confirmed and discarded cases of dengue, chikungunya and Zika viruses in Brazil: A multivariate time-series analysis
Source: PLoS One. 2020 Feb 3;15(2):e0228347. doi: 10.1371/journal.pone.0228347 (PMC6996800; doi:10.1371/journal.pone.0228347)
Supplement: S1 Appendix — VAR model for the 6-dimensional multivariate time series analysis of confirmed and discarded cases of dengue, chikungunya and Zika. (PDF) [file pone.0228347.s008.pdf]

Summary of Regression Results

Model:	VAR
Method:	OLS
Date:	Thu, 26, Dec, 2019
Time:	12:48:20

No. of Equations:	6.00000	BIC:	79.3113
Nobs:	142.000	HQIC:	73.4541
Log likelihood:	-5665.51	FPE:	3.39136e+30
AIC:	69.4447	Det(Omega_mle):	2.38644e+29

Results for equation cases_zika

	coefficient	std. error	t-stat	prob
const	11.094524	28.124098	0.394	0.693
L1.cases_zika	0.897923	0.168115	5.341	0.000
L1.cases_des_zika	1.533060	0.730488	2.099	0.036
L1.cases_chik	0.048381	0.149485	0.324	0.746
L1.cases_des_chik	-0.298380	0.406153	-0.735	0.463
L1.cases_dengue	-0.046167	0.021204	-2.177	0.029
L1.cases_dengue_des	-0.158328	0.049182	-3.219	0.001
L2.cases_zika	-0.690075	0.203275	-3.395	0.001
L2.cases_des_zika	0.943165	0.792161	1.191	0.234
L2.cases_chik	0.020578	0.137753	0.149	0.881
L2.cases_des_chik	0.017230	0.411329	0.042	0.967
L2.cases_dengue	0.136659	0.025724	5.312	0.000
L2.cases_dengue_des	-0.084641	0.057561	-1.470	0.141
L3.cases_zika	-0.211701	0.228697	-0.926	0.355
L3.cases_des_zika	-0.213419	0.762746	-0.280	0.780
L3.cases_chik	-0.153490	0.160796	-0.955	0.340
L3.cases_des_chik	0.854566	0.443824	1.925	0.054
L3.cases_dengue	-0.018555	0.031249	-0.594	0.553
L3.cases_dengue_des	0.085785	0.062131	1.381	0.167
L4.cases_zika	0.036012	0.234096	0.154	0.878
L4.cases_des_zika	0.413401	0.868047	0.476	0.634
L4.cases_chik	-0.172107	0.152852	-1.126	0.260
L4.cases_des_chik	-0.413619	0.462932	-0.893	0.372
L4.cases_dengue	-0.015609	0.028930	-0.540	0.589
L4.cases_dengue_des	0.071332	0.061799	1.154	0.248
L5.cases_zika	0.201610	0.209063	0.964	0.335
L5.cases_des_zika	0.165454	0.822154	0.201	0.841
L5.cases_chik	0.072781	0.154958	0.470	0.639
L5.cases_des_chik	-0.189762	0.459919	-0.413	0.680
L5.cases_dengue	0.015424	0.029177	0.529	0.597
L5.cases_dengue_des	0.002881	0.052582	0.055	0.956
L6.cases_zika	-0.123935	0.227611	-0.545	0.586
L6.cases_des_zika	-1.456457	0.888872	-1.639	0.101
L6.cases_chik	0.091341	0.161122	0.567	0.571
L6.cases_des_chik	-0.271269	0.449494	-0.603	0.546
L6.cases_dengue	0.050604	0.024518	2.064	0.039
L6.cases_dengue_des	0.168810	0.046286	3.647	0.000

L7.cases_zika	-0.417545	0.213913	-1.952	0.051
L7.cases_des_zika	1.985676	0.834852	2.378	0.017
L7.cases_chik	-0.091553	0.169917	-0.539	0.590
L7.cases_des_chik	-0.339353	0.449194	-0.755	0.450
L7.cases_dengue	-0.168528	0.026585	-6.339	0.000
L7.cases_dengue_des	0.053466	0.044131	1.212	0.226
L8.cases_zika	0.304054	0.214296	1.419	0.156
L8.cases_des_zika	0.149818	0.755352	0.198	0.843
L8.cases_chik	0.107014	0.158030	0.677	0.498
L8.cases_des_chik	-0.164668	0.443811	-0.371	0.711
L8.cases_dengue	0.051833	0.029626	1.750	0.080
L8.cases_dengue_des	-0.033110	0.047213	-0.701	0.483
L9.cases_zika	0.054633	0.209230	0.261	0.794
L9.cases_des_zika	-0.967618	0.764804	-1.265	0.206
L9.cases_chik	0.226059	0.156001	1.449	0.147
L9.cases_des_chik	-0.424262	0.452251	-0.938	0.348
L9.cases_dengue	-0.025822	0.030130	-0.857	0.391
L9.cases_dengue_des	0.022919	0.049982	0.459	0.647
L10.cases_zika	-0.080753	0.202077	-0.400	0.689
L10.cases_des_zika	-1.430709	0.727667	-1.966	0.049
L10.cases_chik	-0.225482	0.163548	-1.379	0.168
L10.cases_des_chik	0.550342	0.442219	1.245	0.213
L10.cases_dengue	-0.036137	0.028367	-1.274	0.203
L10.cases_dengue_des	0.098266	0.047316	2.077	0.038
L11.cases_zika	0.628872	0.206281	3.049	0.002
L11.cases_des_zika	-1.458785	0.784026	-1.861	0.063
L11.cases_chik	0.078187	0.163313	0.479	0.632
L11.cases_des_chik	-0.397749	0.447309	-0.889	0.374
L11.cases_dengue	-0.053401	0.027257	-1.959	0.050
L11.cases_dengue_des	0.030319	0.045203	0.671	0.502
L12.cases_zika	-0.401798	0.208689	-1.925	0.054
L12.cases_des_zika	1.027986	0.840143	1.224	0.221
L12.cases_chik	-0.031798	0.151355	-0.210	0.834
L12.cases_des_chik	0.072964	0.412643	0.177	0.860
L12.cases_dengue	0.035607	0.026831	1.327	0.184
L12.cases_dengue_des	0.044942	0.043858	1.025	0.306
L13.cases_zika	-0.197608	0.211541	-0.934	0.350
L13.cases_des_zika	-0.200851	0.862065	-0.233	0.816
L13.cases_chik	0.064924	0.158917	0.409	0.683
L13.cases_des_chik	-0.071529	0.409352	-0.175	0.861
L13.cases_dengue	-0.043492	0.026734	-1.627	0.104
L13.cases_dengue_des	0.110564	0.034900	3.168	0.002

Results for equation cases_des_zika

	coefficient	std. error	t-stat	prob
const	3.500321	6.695504	0.523	0.601
L1.cases_zika	0.153770	0.040023	3.842	0.000
L1.cases_des_zika	0.545798	0.173907	3.138	0.002
L1.cases_chik	-0.002482	0.035588	-0.070	0.944
L1.cases_des_chik	-0.016342	0.096693	-0.169	0.866

L1.cases_dengue	-0.000862	0.005048	-0.171	0.864
L1.cases_dengue_des	-0.058296	0.011709	-4.979	0.000
L2.cases_zika	-0.098163	0.048394	-2.028	0.043
L2.cases_des_zika	0.195727	0.188590	1.038	0.299
L2.cases_chik	0.055188	0.032795	1.683	0.092
L2.cases_des_chik	-0.063797	0.097925	-0.651	0.515
L2.cases_dengue	0.027516	0.006124	4.493	0.000
L2.cases_dengue_des	-0.027617	0.013703	-2.015	0.044
L3.cases_zika	-0.104338	0.054446	-1.916	0.055
L3.cases_des_zika	0.323224	0.181587	1.780	0.075
L3.cases_chik	-0.045744	0.038281	-1.195	0.232
L3.cases_des_chik	0.209283	0.105661	1.981	0.048
L3.cases_dengue	0.002302	0.007439	0.309	0.757
L3.cases_dengue_des	0.002193	0.014791	0.148	0.882
L4.cases_zika	0.041437	0.055731	0.744	0.457
L4.cases_des_zika	-0.143058	0.206656	-0.692	0.489
L4.cases_chik	-0.067515	0.036390	-1.855	0.064
L4.cases_des_chik	0.045655	0.110210	0.414	0.679
L4.cases_dengue	-0.000892	0.006887	-0.129	0.897
L4.cases_dengue_des	0.025749	0.014713	1.750	0.080
L5.cases_zika	0.084788	0.049772	1.704	0.088
L5.cases_des_zika	-0.365668	0.195730	-1.868	0.062
L5.cases_chik	-0.002845	0.036891	-0.077	0.939
L5.cases_des_chik	-0.031000	0.109493	-0.283	0.777
L5.cases_dengue	0.002020	0.006946	0.291	0.771
L5.cases_dengue_des	0.016140	0.012518	1.289	0.197
L6.cases_zika	0.035522	0.054187	0.656	0.512
L6.cases_des_zika	-0.221049	0.211614	-1.045	0.296
L6.cases_chik	-0.007814	0.038358	-0.204	0.839
L6.cases_des_chik	-0.052905	0.107011	-0.494	0.621
L6.cases_dengue	0.005391	0.005837	0.924	0.356
L6.cases_dengue_des	0.034974	0.011019	3.174	0.002
L7.cases_zika	-0.074749	0.050926	-1.468	0.142
L7.cases_des_zika	0.212385	0.198753	1.069	0.285
L7.cases_chik	0.022033	0.040452	0.545	0.586
L7.cases_des_chik	-0.147372	0.106940	-1.378	0.168
L7.cases_dengue	-0.026251	0.006329	-4.148	0.000
L7.cases_dengue_des	0.024216	0.010506	2.305	0.021
L8.cases_zika	0.048191	0.051017	0.945	0.345
L8.cases_des_zika	0.060713	0.179827	0.338	0.736
L8.cases_chik	0.022819	0.037622	0.607	0.544
L8.cases_des_chik	0.050648	0.105658	0.479	0.632
L8.cases_dengue	0.001218	0.007053	0.173	0.863
L8.cases_dengue_des	-0.003618	0.011240	-0.322	0.748
L9.cases_zika	0.011073	0.049811	0.222	0.824
L9.cases_des_zika	-0.209837	0.182077	-1.152	0.249
L9.cases_chik	0.027751	0.037139	0.747	0.455
L9.cases_des_chik	-0.024009	0.107668	-0.223	0.824
L9.cases_dengue	-0.005489	0.007173	-0.765	0.444
L9.cases_dengue_des	0.003254	0.011899	0.273	0.785
L10.cases_zika	0.064279	0.048109	1.336	0.182
L10.cases_des_zika	-0.590941	0.173236	-3.411	0.001
L10.cases_chik	-0.016240	0.038936	-0.417	0.677

L10.cases_des_chik	0.115992	0.105279	1.102	0.271
L10.cases_dengue	-0.009162	0.006753	-1.357	0.175
L10.cases_dengue_des	0.017654	0.011265	1.567	0.117
L11.cases_zika	0.186632	0.049109	3.800	0.000
L11.cases_des_zika	-0.662448	0.186653	-3.549	0.000
L11.cases_chik	0.009343	0.038880	0.240	0.810
L11.cases_des_chik	-0.042072	0.106491	-0.395	0.693
L11.cases_dengue	-0.010161	0.006489	-1.566	0.117
L11.cases_dengue_des	0.018022	0.010761	1.675	0.094
L12.cases_zika	-0.116973	0.049683	-2.354	0.019
L12.cases_des_zika	0.238299	0.200013	1.191	0.233
L12.cases_chik	-0.032113	0.036033	-0.891	0.373
L12.cases_des_chik	0.009633	0.098238	0.098	0.922
L12.cases_dengue	-0.004770	0.006388	-0.747	0.455
L12.cases_dengue_des	0.022606	0.010441	2.165	0.030
L13.cases_zika	-0.002168	0.050362	-0.043	0.966
L13.cases_des_zika	-0.070744	0.205232	-0.345	0.730
L13.cases_chik	-0.009417	0.037833	-0.249	0.803
L13.cases_des_chik	0.106187	0.097454	1.090	0.276
L13.cases_dengue	-0.005714	0.006365	-0.898	0.369
L13.cases_dengue_des	0.013796	0.008309	1.660	0.097

Results for equation cases_chik

	coefficient	std. error	t-stat	prob
const	-13.915982	27.337543	-0.509	0.611
L1.cases_zika	-0.184642	0.163413	-1.130	0.259
L1.cases_des_zika	0.680923	0.710058	0.959	0.338
L1.cases_chik	-0.070040	0.145304	-0.482	0.630
L1.cases_des_chik	-0.651946	0.394794	-1.651	0.099
L1.cases_dengue	0.000021	0.020611	0.001	0.999
L1.cases_dengue_des	0.051685	0.047806	1.081	0.280
L2.cases_zika	-0.074540	0.197590	-0.377	0.706
L2.cases_des_zika	1.260483	0.770006	1.637	0.102
L2.cases_chik	0.712833	0.133901	5.324	0.000
L2.cases_des_chik	-1.693991	0.399825	-4.237	0.000
L2.cases_dengue	-0.050748	0.025005	-2.030	0.042
L2.cases_dengue_des	0.074683	0.055951	1.335	0.182
L3.cases_zika	-0.457288	0.222300	-2.057	0.040
L3.cases_des_zika	0.872903	0.741414	1.177	0.239
L3.cases_chik	0.374834	0.156299	2.398	0.016
L3.cases_des_chik	-1.122729	0.431412	-2.602	0.009
L3.cases_dengue	-0.009599	0.030375	-0.316	0.752
L3.cases_dengue_des	0.117891	0.060393	1.952	0.051
L4.cases_zika	0.180660	0.227549	0.794	0.427
L4.cases_des_zika	-1.596248	0.843770	-1.892	0.059
L4.cases_chik	-0.134500	0.148577	-0.905	0.365
L4.cases_des_chik	0.969467	0.449985	2.154	0.031
L4.cases_dengue	-0.028074	0.028121	-0.998	0.318
L4.cases_dengue_des	0.098683	0.060071	1.643	0.100
L5.cases_zika	-0.432976	0.203216	-2.131	0.033

L5.cases_des_zika	0.181582	0.799161	0.227	0.820
L5.cases_chik	0.090081	0.150624	0.598	0.550
L5.cases_des_chik	-0.761246	0.447056	-1.703	0.089
L5.cases_dengue	-0.015916	0.028361	-0.561	0.575
L5.cases_dengue_des	0.121323	0.051111	2.374	0.018
L6.cases_zika	0.207648	0.221246	0.939	0.348
L6.cases_des_zika	0.245591	0.864013	0.284	0.776
L6.cases_chik	-0.436098	0.156616	-2.785	0.005
L6.cases_des_chik	0.605027	0.436923	1.385	0.166
L6.cases_dengue	-0.074590	0.023832	-3.130	0.002
L6.cases_dengue_des	0.103928	0.044992	2.310	0.021
L7.cases_zika	-0.364973	0.207931	-1.755	0.079
L7.cases_des_zika	0.416176	0.811503	0.513	0.608
L7.cases_chik	0.247243	0.165165	1.497	0.134
L7.cases_des_chik	-0.358135	0.436632	-0.820	0.412
L7.cases_dengue	-0.014547	0.025841	-0.563	0.573
L7.cases_dengue_des	0.096209	0.042897	2.243	0.025
L8.cases_zika	-0.103750	0.208303	-0.498	0.618
L8.cases_des_zika	-0.667996	0.734227	-0.910	0.363
L8.cases_chik	-0.269907	0.153610	-1.757	0.079
L8.cases_des_chik	1.534154	0.431399	3.556	0.000
L8.cases_dengue	-0.068830	0.028797	-2.390	0.017
L8.cases_dengue_des	0.164204	0.045893	3.578	0.000
L9.cases_zika	-0.001881	0.203378	-0.009	0.993
L9.cases_des_zika	-0.436446	0.743415	-0.587	0.557
L9.cases_chik	-0.214708	0.151638	-1.416	0.157
L9.cases_des_chik	0.915687	0.439603	2.083	0.037
L9.cases_dengue	-0.066114	0.029288	-2.257	0.024
L9.cases_dengue_des	0.115287	0.048584	2.373	0.018
L10.cases_zika	0.150883	0.196426	0.768	0.442
L10.cases_des_zika	-0.380277	0.707316	-0.538	0.591
L10.cases_chik	0.098955	0.158974	0.622	0.534
L10.cases_des_chik	0.897270	0.429851	2.087	0.037
L10.cases_dengue	-0.050719	0.027574	-1.839	0.066
L10.cases_dengue_des	0.097272	0.045993	2.115	0.034
L11.cases_zika	-0.112929	0.200512	-0.563	0.573
L11.cases_des_zika	-0.069715	0.762098	-0.091	0.927
L11.cases_chik	0.098362	0.158746	0.620	0.536
L11.cases_des_chik	-0.245954	0.434799	-0.566	0.572
L11.cases_dengue	-0.038058	0.026494	-1.436	0.151
L11.cases_dengue_des	0.129383	0.043939	2.945	0.003
L12.cases_zika	-0.306173	0.202853	-1.509	0.131
L12.cases_des_zika	0.752396	0.816646	0.921	0.357
L12.cases_chik	-0.419601	0.147122	-2.852	0.004
L12.cases_des_chik	0.833122	0.401102	2.077	0.038
L12.cases_dengue	-0.075604	0.026081	-2.899	0.004
L12.cases_dengue_des	0.092518	0.042632	2.170	0.030
L13.cases_zika	0.114706	0.205625	0.558	0.577
L13.cases_des_zika	-0.213096	0.837955	-0.254	0.799
L13.cases_chik	-0.236499	0.154472	-1.531	0.126
L13.cases_des_chik	0.575234	0.397904	1.446	0.148
L13.cases_dengue	-0.010782	0.025987	-0.415	0.678
L13.cases_dengue_des	0.023736	0.033924	0.700	0.484

==

Results for equation cases_des_chik

==

	coefficient	std. error	t-stat	prob
const	1.283177	11.173407	0.115	0.909
L1.cases_zika	-0.035587	0.066790	-0.533	0.594
L1.cases_des_zika	0.381725	0.290215	1.315	0.188
L1.cases_chik	-0.063587	0.059389	-1.071	0.284
L1.cases_des_chik	-0.042706	0.161360	-0.265	0.791
L1.cases_dengue	0.005938	0.008424	0.705	0.481
L1.cases_dengue_des	-0.015892	0.019539	-0.813	0.416
L2.cases_zika	-0.094376	0.080759	-1.169	0.243
L2.cases_des_zika	0.244900	0.314717	0.778	0.436
L2.cases_chik	0.105473	0.054728	1.927	0.054
L2.cases_des_chik	-0.266636	0.163417	-1.632	0.103
L2.cases_dengue	-0.003935	0.010220	-0.385	0.700
L2.cases_dengue_des	-0.001426	0.022868	-0.062	0.950
L3.cases_zika	-0.125927	0.090859	-1.386	0.166
L3.cases_des_zika	0.880798	0.303031	2.907	0.004
L3.cases_chik	0.002108	0.063882	0.033	0.974
L3.cases_des_chik	-0.468757	0.176327	-2.658	0.008
L3.cases_dengue	0.000310	0.012415	0.025	0.980
L3.cases_dengue_des	0.014819	0.024684	0.600	0.548
L4.cases_zika	0.035549	0.093004	0.382	0.702
L4.cases_des_zika	-0.268716	0.344866	-0.779	0.436
L4.cases_chik	-0.016094	0.060727	-0.265	0.791
L4.cases_des_chik	-0.063439	0.183918	-0.345	0.730
L4.cases_dengue	-0.003803	0.011493	-0.331	0.741
L4.cases_dengue_des	0.039624	0.024552	1.614	0.107
L5.cases_zika	-0.136398	0.083059	-1.642	0.101
L5.cases_des_zika	-0.495480	0.326633	-1.517	0.129
L5.cases_chik	0.193498	0.061563	3.143	0.002
L5.cases_des_chik	-0.398090	0.182721	-2.179	0.029
L5.cases_dengue	0.000434	0.011592	0.037	0.970
L5.cases_dengue_des	0.071603	0.020890	3.428	0.001
L6.cases_zika	0.090106	0.090428	0.996	0.319
L6.cases_des_zika	0.006465	0.353140	0.018	0.985
L6.cases_chik	-0.041019	0.064012	-0.641	0.522
L6.cases_des_chik	-0.000499	0.178579	-0.003	0.998
L6.cases_dengue	-0.038489	0.009741	-3.951	0.000
L6.cases_dengue_des	0.053060	0.018389	2.885	0.004
L7.cases_zika	-0.018830	0.084985	-0.222	0.825
L7.cases_des_zika	0.003748	0.331678	0.011	0.991
L7.cases_chik	0.069117	0.067506	1.024	0.306
L7.cases_des_chik	-0.130023	0.178460	-0.729	0.466
L7.cases_dengue	-0.007088	0.010562	-0.671	0.502
L7.cases_dengue_des	0.012797	0.017533	0.730	0.465
L8.cases_zika	-0.046181	0.085137	-0.542	0.588
L8.cases_des_zika	0.101719	0.300094	0.339	0.735
L8.cases_chik	-0.005318	0.062784	-0.085	0.932
L8.cases_des_chik	0.366547	0.176322	2.079	0.038

L8.cases_dengue	-0.007598	0.011770	-0.646	0.519
L8.cases_dengue_des	0.032905	0.018757	1.754	0.079
L9.cases_zika	-0.087432	0.083125	-1.052	0.293
L9.cases_des_zika	-0.114796	0.303849	-0.378	0.706
L9.cases_chik	-0.067650	0.061978	-1.092	0.275
L9.cases_des_chik	0.267229	0.179675	1.487	0.137
L9.cases_dengue	-0.028796	0.011970	-2.406	0.016
L9.cases_dengue_des	0.048682	0.019857	2.452	0.014
L10.cases_zika	0.009379	0.080283	0.117	0.907
L10.cases_des_zika	-0.017948	0.289095	-0.062	0.950
L10.cases_chik	-0.079265	0.064976	-1.220	0.222
L10.cases_des_chik	0.182226	0.175689	1.037	0.300
L10.cases_dengue	-0.024145	0.011270	-2.142	0.032
L10.cases_dengue_des	0.031336	0.018798	1.667	0.096
L11.cases_zika	0.125035	0.081953	1.526	0.127
L11.cases_des_zika	-0.517683	0.311485	-1.662	0.097
L11.cases_chik	0.004791	0.064883	0.074	0.941
L11.cases_des_chik	0.050466	0.177711	0.284	0.776
L11.cases_dengue	-0.003391	0.010829	-0.313	0.754
L11.cases_dengue_des	0.039186	0.017959	2.182	0.029
L12.cases_zika	-0.086502	0.082910	-1.043	0.297
L12.cases_des_zika	0.084077	0.333780	0.252	0.801
L12.cases_chik	-0.039633	0.060132	-0.659	0.510
L12.cases_des_chik	0.140431	0.163939	0.857	0.392
L12.cases_dengue	-0.025657	0.010660	-2.407	0.016
L12.cases_dengue_des	0.039357	0.017424	2.259	0.024
L13.cases_zika	-0.005186	0.084043	-0.062	0.951
L13.cases_des_zika	0.213569	0.342489	0.624	0.533
L13.cases_chik	-0.035577	0.063136	-0.564	0.573
L13.cases_des_chik	0.090083	0.162631	0.554	0.580
L13.cases_dengue	-0.002852	0.010621	-0.269	0.788
L13.cases_dengue_des	0.005171	0.013865	0.373	0.709

Results for equation cases_dengue

	coefficient	std. error	t-stat	prob
const	-160.671309	190.319635	-0.844	0.399
L1.cases_zika	-0.446867	1.137659	-0.393	0.694
L1.cases_des_zika	17.070065	4.943314	3.453	0.001
L1.cases_chik	0.395457	1.011584	0.391	0.696
L1.cases_des_chik	-2.921603	2.748492	-1.063	0.288
L1.cases_dengue	0.016670	0.143491	0.116	0.908
L1.cases_dengue_des	-0.673699	0.332818	-2.024	0.043
L2.cases_zika	-0.585493	1.375590	-0.426	0.670
L2.cases_des_zika	-5.631557	5.360661	-1.051	0.293
L2.cases_chik	-0.412694	0.932197	-0.443	0.658
L2.cases_des_chik	2.864799	2.783518	1.029	0.303
L2.cases_dengue	0.887117	0.174081	5.096	0.000
L2.cases_dengue_des	-0.571866	0.389521	-1.468	0.142
L3.cases_zika	-6.004440	1.547621	-3.880	0.000
L3.cases_des_zika	13.362043	5.161604	2.589	0.010

L3.cases_chik	-1.186516	1.088126	-1.090	0.276
L3.cases_des_chik	-0.487387	3.003420	-0.162	0.871
L3.cases_dengue	0.308792	0.211466	1.460	0.144
L3.cases_dengue_des	0.490396	0.420446	1.166	0.243
L4.cases_zika	0.298268	1.584157	0.188	0.851
L4.cases_des_zika	3.009798	5.874192	0.512	0.608
L4.cases_chik	-0.426530	1.034371	-0.412	0.680
L4.cases_des_chik	0.098009	3.132727	0.031	0.975
L4.cases_dengue	-0.170064	0.195771	-0.869	0.385
L4.cases_dengue_des	0.585632	0.418203	1.400	0.161
L5.cases_zika	3.951725	1.414760	2.793	0.005
L5.cases_des_zika	-13.656467	5.563630	-2.455	0.014
L5.cases_chik	1.583714	1.048621	1.510	0.131
L5.cases_des_chik	-4.127235	3.112334	-1.326	0.185
L5.cases_dengue	-0.043430	0.197442	-0.220	0.826
L5.cases_dengue_des	0.446266	0.355828	1.254	0.210
L6.cases_zika	0.447195	1.540278	0.290	0.772
L6.cases_des_zika	-5.129775	6.015122	-0.853	0.394
L6.cases_chik	-0.008736	1.090334	-0.008	0.994
L6.cases_des_chik	1.313012	3.041789	0.432	0.666
L6.cases_dengue	0.058863	0.165918	0.355	0.723
L6.cases_dengue_des	0.444011	0.313226	1.418	0.156
L7.cases_zika	-1.817495	1.447579	-1.256	0.209
L7.cases_des_zika	4.443436	5.649558	0.787	0.432
L7.cases_chik	-0.113654	1.149852	-0.099	0.921
L7.cases_des_chik	-1.348182	3.039760	-0.444	0.657
L7.cases_dengue	-0.316527	0.179903	-1.759	0.079
L7.cases_dengue_des	-0.318393	0.298639	-1.066	0.286
L8.cases_zika	-0.528749	1.450170	-0.365	0.715
L8.cases_des_zika	12.391995	5.111574	2.424	0.015
L8.cases_chik	0.085895	1.069411	0.080	0.936
L8.cases_des_chik	-0.622599	3.003333	-0.207	0.836
L8.cases_dengue	0.268022	0.200480	1.337	0.181
L8.cases_dengue_des	-0.660131	0.319498	-2.066	0.039
L9.cases_zika	-0.333280	1.415886	-0.235	0.814
L9.cases_des_zika	0.307700	5.175534	0.059	0.953
L9.cases_chik	0.356858	1.055681	0.338	0.735
L9.cases_des_chik	0.542103	3.060448	0.177	0.859
L9.cases_dengue	-0.186561	0.203895	-0.915	0.360
L9.cases_dengue_des	-0.236778	0.338235	-0.700	0.484
L10.cases_zika	-0.408625	1.367484	-0.299	0.765
L10.cases_des_zika	0.467138	4.924224	0.095	0.924
L10.cases_chik	-0.388443	1.106751	-0.351	0.726
L10.cases_des_chik	0.715099	2.992557	0.239	0.811
L10.cases_dengue	-0.244566	0.191965	-1.274	0.203
L10.cases_dengue_des	-0.004361	0.320193	-0.014	0.989
L11.cases_zika	2.647472	1.395934	1.897	0.058
L11.cases_des_zika	-15.606905	5.305609	-2.942	0.003
L11.cases_chik	-1.134750	1.105162	-1.027	0.305
L11.cases_des_chik	2.730199	3.027003	0.902	0.367
L11.cases_dengue	-0.141452	0.184450	-0.767	0.443
L11.cases_dengue_des	0.538952	0.305895	1.762	0.078
L12.cases_zika	-4.326466	1.412230	-3.064	0.002

L12.cases_des_zika	17.269488	5.685362	3.038	0.002
L12.cases_chik	0.221794	1.024242	0.217	0.829
L12.cases_des_chik	-2.929602	2.792410	-1.049	0.294
L12.cases_dengue	-0.142483	0.181570	-0.785	0.433
L12.cases_dengue_des	0.354326	0.296796	1.194	0.233
L13.cases_zika	-1.819899	1.431527	-1.271	0.204
L13.cases_des_zika	4.667516	5.833712	0.800	0.424
L13.cases_chik	0.408421	1.075411	0.380	0.704
L13.cases_des_chik	-1.187314	2.770142	-0.429	0.668
L13.cases_dengue	0.094118	0.180914	0.520	0.603
L13.cases_dengue_des	0.495132	0.236171	2.096	0.036

Results for equation cases_dengue_des

	coefficient	std. error	t-stat	prob
const	-46.353323	98.349311	-0.471	0.637
L1.cases_zika	0.177450	0.587895	0.302	0.763
L1.cases_des_zika	2.444867	2.554500	0.957	0.339
L1.cases_chik	0.107638	0.522745	0.206	0.837
L1.cases_des_chik	1.639880	1.420307	1.155	0.248
L1.cases_dengue	0.250785	0.074150	3.382	0.001
L1.cases_dengue_des	-0.692674	0.171987	-4.027	0.000
L2.cases_zika	1.725036	0.710848	2.427	0.015
L2.cases_des_zika	-0.054749	2.770168	-0.020	0.984
L2.cases_chik	-0.568567	0.481721	-1.180	0.238
L2.cases_des_chik	1.667821	1.438407	1.159	0.246
L2.cases_dengue	0.273475	0.089958	3.040	0.002
L2.cases_dengue_des	-0.745366	0.201288	-3.703	0.000
L3.cases_zika	-3.770326	0.799746	-4.714	0.000
L3.cases_des_zika	10.966172	2.667304	4.111	0.000
L3.cases_chik	-0.285550	0.562299	-0.508	0.612
L3.cases_des_chik	0.085305	1.552043	0.055	0.956
L3.cases_dengue	0.209278	0.109277	1.915	0.055
L3.cases_dengue_des	0.049734	0.217269	0.229	0.819
L4.cases_zika	0.992174	0.818627	1.212	0.226
L4.cases_des_zika	-0.894029	3.035540	-0.295	0.768
L4.cases_chik	-0.026598	0.534520	-0.050	0.960
L4.cases_des_chik	0.976526	1.618863	0.603	0.546
L4.cases_dengue	0.087443	0.101166	0.864	0.387
L4.cases_dengue_des	0.177211	0.216110	0.820	0.412
L5.cases_zika	-0.232007	0.731089	-0.317	0.751
L5.cases_des_zika	-6.327113	2.875054	-2.201	0.028
L5.cases_chik	0.050366	0.541884	0.093	0.926
L5.cases_des_chik	-0.541035	1.608326	-0.336	0.737
L5.cases_dengue	0.039690	0.102030	0.389	0.697
L5.cases_dengue_des	0.219223	0.183877	1.192	0.233
L6.cases_zika	1.340859	0.795952	1.685	0.092
L6.cases_des_zika	-0.994711	3.108366	-0.320	0.749
L6.cases_chik	-0.939093	0.563440	-1.667	0.096
L6.cases_des_chik	2.636285	1.571871	1.677	0.094
L6.cases_dengue	0.014518	0.085740	0.169	0.866

L6.cases_dengue_des	0.136015	0.161862	0.840	0.401
L7.cases_zika	-0.764867	0.748049	-1.022	0.307
L7.cases_des_zika	4.145726	2.919458	1.420	0.156
L7.cases_chik	0.191633	0.594196	0.323	0.747
L7.cases_des_chik	-2.489073	1.570822	-1.585	0.113
L7.cases_dengue	-0.051599	0.092967	-0.555	0.579
L7.cases_dengue_des	-0.089853	0.154324	-0.582	0.560
L8.cases_zika	-0.090293	0.749388	-0.120	0.904
L8.cases_des_zika	5.617012	2.641450	2.126	0.033
L8.cases_chik	-0.329741	0.552627	-0.597	0.551
L8.cases_des_chik	1.354375	1.551998	0.873	0.383
L8.cases_dengue	-0.035550	0.103600	-0.343	0.731
L8.cases_dengue_des	-0.166102	0.165103	-1.006	0.314
L9.cases_zika	-0.976949	0.731671	-1.335	0.182
L9.cases_des_zika	-1.514698	2.674502	-0.566	0.571
L9.cases_chik	0.959388	0.545532	1.759	0.079
L9.cases_des_chik	-0.888185	1.581513	-0.562	0.574
L9.cases_dengue	0.022566	0.105364	0.214	0.830
L9.cases_dengue_des	0.086052	0.174786	0.492	0.622
L10.cases_zika	-0.764555	0.706659	-1.082	0.279
L10.cases_des_zika	0.532113	2.544635	0.209	0.834
L10.cases_chik	-0.432713	0.571923	-0.757	0.449
L10.cases_des_chik	1.046211	1.546430	0.677	0.499
L10.cases_dengue	-0.122769	0.099200	-1.238	0.216
L10.cases_dengue_des	-0.032263	0.165463	-0.195	0.845
L11.cases_zika	2.179960	0.721361	3.022	0.003
L11.cases_des_zika	-10.306922	2.741719	-3.759	0.000
L11.cases_chik	-0.429197	0.571102	-0.752	0.452
L11.cases_des_chik	1.078881	1.564230	0.690	0.490
L11.cases_dengue	-0.004526	0.095316	-0.047	0.962
L11.cases_dengue_des	0.194530	0.158074	1.231	0.218
L12.cases_zika	-1.665553	0.729782	-2.282	0.022
L12.cases_des_zika	8.374264	2.937960	2.850	0.004
L12.cases_chik	-0.065857	0.529286	-0.124	0.901
L12.cases_des_chik	-0.944793	1.443002	-0.655	0.513
L12.cases_dengue	-0.099250	0.093828	-1.058	0.290
L12.cases_dengue_des	0.086088	0.153372	0.561	0.575
L13.cases_zika	-1.138808	0.739754	-1.539	0.124
L13.cases_des_zika	1.008716	3.014621	0.335	0.738
L13.cases_chik	-0.396625	0.555728	-0.714	0.475
L13.cases_des_chik	0.241946	1.431495	0.169	0.866
L13.cases_dengue	-0.029184	0.093489	-0.312	0.755
L13.cases_dengue_des	0.277434	0.122044	2.273	0.023

==
