# Supplementary material for: Interdependence between confirmed and discarded cases of dengue, chikungunya and Zika viruses in Brazil: A multivariate time-series analysis
Source: PLoS One. 2020 Feb 3;15(2):e0228347. doi: 10.1371/journal.pone.0228347 (PMC6996800; doi:10.1371/journal.pone.0228347)
Supplement: S2 Appendix — (DOC) [file pone.0228347.s009.doc]

STROBE Statement—checklist of items that should be included in reports of observational studies

|  | Item No | Recommendation |
| --- | --- | --- |
| **Title and abstract** | 1 | (*a*) Indicate the study’s design with a commonly used term in the title or the abstract:  ***“Interdependence between confirmed and discarded cases of dengue, chikungunya and Zika viruses in Brazil: A multivariate time-series analysis”*** |
|  |  | (*b*) Provide in the abstract an informative and balanced summary of what was done and what was found:  “***Background:*** “The co-circulation of different arboviruses in the same time and space poses a significant threat to public health given their rapid geographic dispersion and serious health, social, and economic impact. Therefore, it is crucial to have high quality of case registration to estimate the real impact of each arboviruses in the population.”  “***Objective:*** “In this work, a Vector Autoregressive (VAR) model was developed to investigate the interrelationships between discarded and confirmed cases of dengue, chikungunya, and Zika in Brazil”  “***Setting:*** “We used data from the Brazilian National Notifiable Diseases Information System (SINAN) from 2010 to 2017.”  “***Results:*** “There were three peaks in the series of dengue notification in this period  occurring in 2013, 2015 and in 2016. The series of reported cases of both Zika and  chikungunya reached their peak in late 2015 and early 2016. The VAR model shows  that the Zika series have a significant impact on the dengue series and vice versa,  suggesting that several discarded and confirmed cases of dengue could actually have been cases of Zika. The model also suggests that the series of confirmed and discarded chikungunya cases are almost independent of the cases of Zika, however, affecting the series of dengue.”  “***Conclusions:*** “In conclusion, co-circulation of arboviruses with similar symptoms  could have lead to misdiagnosed diseases in the surveillance system. We argue that the routinely use of mathematical and statistical models in association with traditional symptom-surveillance could help to decrease such errors and to provide early indication of possible future outbreaks. These findings address the challenges regarding notification biases and shed new light on how to handle reported cases based only in clinical-epidemiological criteria when multiples arboviruses co-circulate in the same population.” |
| Introduction | | |
| Background/rationale | 2 | Explain the scientific background and rationale for the investigation being reported:  ***“Introduction, paragraph 1 and 2”*** |
| Objectives | 3 | State specific objectives, including any prespecified hypotheses:  ***“Introduction, paragraph 3”*** |
| Methods | | |
| Study design | 4 | Present key elements of study design early in the paper |
| Setting | 5 | Describe the setting, locations, and relevant dates, including periods of recruitment, exposure, follow-up, and data collection:  ***“Methods, subsection entitled 'Data source’ ”*** |
| Participants | 6 | (*a*) *Cohort study*—Give the eligibility criteria, and the sources and methods of selection of participants. Describe methods of follow-up  *Case-control study*—Give the eligibility criteria, and the sources and methods of case ascertainment and control selection. Give the rationale for the choice of cases and controls  *Cross-sectional study*—Give the eligibility criteria, and the sources and methods of selection of participants  ***“Methods, subsection entitled 'Data source’ ”*** |
|  |  | (*b*) *Cohort study*—For matched studies, give matching criteria and number of exposed and unexposed  *Case-control study*—For matched studies, give matching criteria and the number of controls per case |
| Variables | 7 | Clearly define all outcomes, exposures, predictors, potential confounders, and effect modifiers. Give diagnostic criteria, if applicable  ***“Methods, subsection entitled 'Data source’ ”*** |
| Data sources/ measurement | 8* | For each variable of interest, give sources of data and details of methods of assessment (measurement). Describe comparability of assessment methods if there is more than one group |
| Bias | 9 | Describe any efforts to address potential sources of bias  ***“Methods, subsection entitled 'Data source’ ”*** |
| Study size | 10 | Explain how the study size was arrived at  ***“The number of cases in the area during the study period determined the sample size.””*** |
| Quantitative variables | 11 | Explain how quantitative variables were handled in the analyses. If applicable, describe which groupings were chosen and why |
| Statistical methods | 12 | (*a*) Describe all statistical methods, including those used to control for confounding  ***“Methods, subsection entitled 'Multivariate time-series analysis’ ”*** |
|  |  | (*b*) Describe any methods used to examine subgroups and interactions  ***“Methods, subsection entitled 'Multivariate time-series analysis’ ”*** |
|  |  | (*c*) Explain how missing data were addressed |
|  |  | (*d*) *Cohort study*—If applicable, explain how loss to follow-up was addressed  *Case-control study*—If applicable, explain how matching of cases and controls was addressed  *Cross-sectional study*—If applicable, describe analytical methods taking account of sampling strategy  ***“Methods, subsection entitled 'Multivariate time-series analysis’ ”*** |
|  |  | (*e*) Describe any sensitivity analyses |

Continued on next page

| Results | | |
| --- | --- | --- |
| Participants | 13* | (a) Report numbers of individuals at each stage of study—eg numbers potentially eligible, examined for eligibility, confirmed eligible, included in the study, completing follow-up, and analysed  ***“Results, paragraph 1 and 2”*** |
|  |  | (b) Give reasons for non-participation at each stage |
|  |  | (c) Consider use of a flow diagram |
| Descriptive data | 14* | (a) Give characteristics of study participants (eg demographic, clinical, social) and information on exposures and potential confounders  ***“Results, paragraph 1 and 2”*** |
|  |  | (b) Indicate number of participants with missing data for each variable of interest  ***“Not applicable”*** |
|  |  | (c) *Cohort study*—Summarise follow-up time (eg, average and total amount)  ***“Results, paragraph 1 and 2”*** |
| Outcome data | 15* | *Cohort study*—Report numbers of outcome events or summary measures over time |
|  |  | *Case-control study—*Report numbers in each exposure category, or summary measures of exposure |
|  |  | *Cross-sectional study—*Report numbers of outcome events or summary measures |
| Main results | 16 | (*a*) Give unadjusted estimates and, if applicable, confounder-adjusted estimates and their precision (eg, 95% confidence interval). Make clear which confounders were adjusted for and why they were included  ***“Results, paragraph 3 - 14”*** |
|  |  | (*b*) Report category boundaries when continuous variables were categorized |
|  |  | (*c*) If relevant, consider translating estimates of relative risk into absolute risk for a meaningful time period |
| Other analyses | 17 | Report other analyses done—eg analyses of subgroups and interactions, and sensitivity analyses |
| Discussion | | |
| Key results | 18 | Summarise key results with reference to study objectives  ***“Discussion, paragraph 1-4”*** |
| Limitations | 19 | Discuss limitations of the study, taking into account sources of potential bias or imprecision. Discuss both direction and magnitude of any potential bias  ***“Discussion, paragraph 7and 8”*** |
| Interpretation | 20 | Give a cautious overall interpretation of results considering objectives, limitations, multiplicity of analyses, results from similar studies, and other relevant evidence  ***“Discussion, paragraph 5-6 and 9-10”*** |
| Generalisability | 21 | Discuss the generalisability (external validity) of the study results  ***“Discussion, paragraph 11”*** |
| Other information | | |
| Funding | 22 | Give the source of funding and the role of the funders for the present study and, if applicable, for the original study on which the present article is based  **“This work is supported by the Center of Data and Knowledge Integration for Health (CIDACS) through the Zika Platform- a long-term surveillance platform for the Zika virus and microcephaly, Unified Health System (SUS) - Brazilian Ministry of Health.”** |

*Give information separately for cases and controls in case-control studies and, if applicable, for exposed and unexposed groups in cohort and cross-sectional studies.

**Note:** An Explanation and Elaboration article discusses each checklist item and gives methodological background and published examples of transparent reporting. The STROBE checklist is best used in conjunction with this article (freely available on the Web sites of PLoS Medicine at http://www.plosmedicine.org/, Annals of Internal Medicine at http://www.annals.org/, and Epidemiology at http://www.epidem.com/). Information on the STROBE Initiative is available at www.strobe-statement.org.
